# Endothelial Protein C Receptor (EPCR), Protease Activated Receptor-1 (PAR-1) and Their Interplay in Cancer Growth and Metastatic Dissemination

**DOI:** 10.3390/cancers11010051

**Published:** 2019-01-08

**Authors:** Marek Z. Wojtukiewicz, Dominika Hempel, Ewa Sierko, Stephanie C. Tucker, Kenneth V. Honn

**Affiliations:** 1 Department of Oncology, Medical University of Bialystok, 15-089 Bialystok, Poland; domhem@wp.pl (D.H.); ewa.sierko@iq.pl (E.S.); 2 Department of Clinical Oncology, Comprehensive Cancer Center in Bialystok, 15-027 Bialystok, Poland; 3 Department of Radiotherapy, Comprehensive Cancer Center in Bialystok, 15-027 Bialystok, Poland; 4 Bioactive Lipids Research Program, Department of Pathology-School of Medicine, Wayne State University, Detroit, MI 48202, USA; stucker@med.wayne.edu; 5 Karmanos Cancer Institute, Detroit, MI 48201, USA; k.v.honn@wayne.edu; 6 Department of Chemistry, Wayne State University, Detroit, MI 48202, USA; 7 Department of Oncology, Wayne State University, Detroit, MI 48202, USA

**Keywords:** EPCR, APC, PAR-1, cancer, metastasis

## Abstract

Endothelial protein C receptor (EPCR) and protease activated receptor 1 (PAR-1) by themselves play important role in cancer growth and dissemination. Moreover, interactions between the two receptors are essential for tumor progression. EPCR is a cell surface transmembrane glycoprotein localized predominantly on endothelial cells (ECs). It is a vital component of the activated protein C (APC)—mediated anticoagulant and cytoprotective signaling cascade. PAR-1, which belongs to a family of G protein–coupled cell surface receptors, is also widely distributed on endothelial and blood cells, where it plays a critical role in hemostasis. Both EPCR and PAR-1, generally considered coagulation-related receptors, are implicated in carcinogenesis and dissemination of diverse tumor types, and their expression correlates with clinical outcome of cancer patients. Existing data explain some mechanisms by which EPCR/PAR-1 affects cancer growth and metastasis; however, the exact molecular basis of cancer invasion associated with the signaling is still obscure. Here, we discuss the role of EPCR and PAR-1 reciprocal interactions in cancer progression as well as potential therapeutic options targeted specifically to interact with EPCR/PAR-1-induced signaling in cancer patients.

## 1. Introduction

Since the landmark publications by Bouillaud and Trousseau, presenting the relationship between coagulation and cancer, numerous studies have been performed to elucidate the exact mechanisms by which the procoagulant phenotype might promote the dissemination of malignant cells. It is now clear that the protease activated cell surface transmembrane receptors, endothelial protein C receptor (EPCR) and protease activated receptor 1 (PAR-1), apart from their roles in hemostasis and inflammation, influence cancer biology [1,2,3]. These receptors have been demonstrated to promote cancer invasion and metastasis in part by activating antiapoptotic pathways as well as to facilitate tumor cell migration, proliferation, angiogenesis and interactions with host vascular cells. 

PAR-1, is a thrombin-activated receptor of a specific pathway of activation with proteolytic cleavage of the N-terminus. Interestingly, thrombin-mediated PAR-1 activation in ECs may be modulated by interactions with the EPCR, thereby inducing alternate cascades of intracellular events. That is to say, thrombin-activated protein C (PC) in complex with EPCR and co-factor thrombomodulin (TM), may activate PAR-1 localized on ECs to exert anticoagulant, anti-inflammatory, and crucially for cancer biology, cytoprotective effects. That is why the interactions between EPCR and PAR-1 have become the focus of research in terms of tumor progression. The APC/EPCR/PAR-1 pathway induces motility and proliferation of ECs as well as promotes angiogenesis via vascular-protective signaling and tube formation, thereby facilitating cancer dissemination [4]. The interaction axis of EPCR/thrombin/PAR-1 on endothelial cells represents an attractive target for inhibiting metastasis, as our previous studies with α-thrombin demonstrated that the enzyme increases adhesion of W256 carcinoma cells to rat aortic ECs and fibronectin by 50–300% [5,6]. Thrombin mediated EPCR/PAR-1 interactions are also observed in bone marrow where these proteins regulate repopulation, survival and chemoresistance of blood-forming progenitor cells by regulating NO production [7,8]. Furthermore, EPCR and PAR-1 are co-expressed in gastric cancer (GC) cells, where EPCR exerts pro-carcinogenic effects by inducing PAR-1-dependent ERK1/2-MAPK pathway signaling to ultimately regulate proliferation and migration of tumor cells. Another hypothesis posits that differences in duration of PAR-1 signaling in endothelial cells may also influence signaling specificity of coagulation proteases [9].

On the other hand, there are data that APC-mediated EPCR activation triggers cytoprotective signaling cascades resulting in vascular barrier enhancement, reduced cancer cell extravasation and inhibition of metastasis [10,11]. The role of the APC/EPCR signaling pathway in limiting cancer cell metastasis may relate to the findings that vitamin K antagonists (VKA) were ineffective in cancer patients in the clinical setting [12]. The conflicting data may result from the fact that in tumor cells expressing both receptors the final biological effects depends on reciprocal interaction influenced by a variety of ligands and cofactors like PC and its inhibitor (PCI), plasminogen activator inhibitor (PAI)-1, thrombin, thrombomodulin (TM), sphingosine 1 and 3 phosphate receptor S1P1, S1P3 and procoagulants, e.g., tissue factor (TF) [13]. Additionally, factor VIIa (FVIIa) modulates thrombin-mediated as well as EPCR-elicited PAR-1 activation in ECs in a likely complex with APC [14]. There are recognized signaling pathways elicited by the APC/EPCR/PAR-1 axis that are independent of protease cleavage sites, which may reconcile some conflicting data in this area [15].

The EPCR-dependent effects of APC as well as the role of PAR-1 in the biology of cancer have been separate topics of recent reviews [1,2,16]. Here, we present the newest knowledge regarding EPCR and PAR-1 reciprocal interactions in cancer progression as well as potential therapeutic options targeted specifically to inhibit or activate EPCR/PAR-1 -induced signaling in cancer patients.

## 2. EPCR and Cancer

EPCR is a type 1 transmembrane protein expressed prominently on the endothelium of the large vessels, but can also be observed on dendritic cells, leukocytes (lymphocytes, monocytes, neutrophils), epithelial cells, keratinocytes, pneumocytes, cardiomyocytes, chondrocytes and osteoblasts [17]. Interestingly EPCR has been described on hematopoietic stem cells and its expression decreases when cells become lineage specific [18] indicating that EPCR plays a potential role in stem cell proliferation and differentiation [19]. Additionally, EPCR can be cleaved from the cell surface by matrix metalloproteinases (MMPs) and circulate as a soluble form (sEPCR) in the blood where it still may bind to PC and APC, as well as interact with integrins on neutrophils during sepsis. Soluble EPCR may alter the active site of APC making phospholipid interactions impossible, thus blocking APC anticoagulant function.

EPCR expression is regulated by multiple factors, e.g., transcription is suppressed by lipopolysaccharide, IL-1β, TNFα, and thrombin. There are gene polymorphisms of EPCR, and haplotype A3 is reportedly responsible for elevated levels of plasma EPCR in the course of inflammatory disorders like sepsis or lupus erythematosus [20,21,22]. Moreover, haplotype A3-mediated elevation of sEPCR has been associated with elevated markers of prothrombin activation (fragment 1–2), venous thrombosis, unexplained fetal death [23] and elevated risk of coronary diseases [24,25,26]. Patients with haplotype 1 EPCR had lower levels of sEPCR and reduced risk of venous thromboembolism. Polymorphism of EPCR may have significance in the biology of cancer as oncology patients commonly have an increased thrombotic state. Apart from haplotypes of EPCR, a single nucleotide polymorphism (SNP) of EPCR, rs2069948, was associated with estrogen receptor (ER) and progesterone receptor (PR) positivity in breast cancer specimens [27]. 

**Figure 1:**
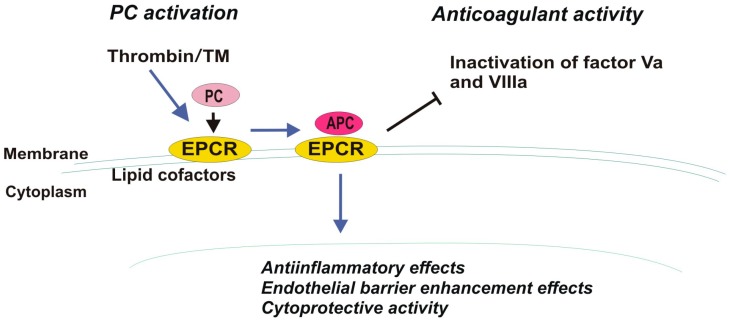
Protein C (PC) activation mediated by thrombin, thrombomodulin (TM) and EPCR (endothelial protein C receptor) and its diverse biological functions (anticoagulant and cellular effects).

The structure of the EPCR, confirmed via crystallization, is similar to the major histocompatibility complex class 1/CDI family of proteins, which are commonly involved in immunity, which partially explains the contribution of EPCR to inflammation. EPCR is located in the caveolin-positive lipid compartment of the membrane close to thrombomodulin (TM) [28]. The cytoplasmic tail (Arg-Arg-Cys-COOH) of EPCR, which does not play a direct role in cell signaling, is likely essential for anchoring EPCR into lipid rafts near the source of signal mediators [2]. The extracellular domain of EPCR is comprised of two α-helices that expose residues that react with vitamin K-dependent liver plasma glycoprotein PC or APC [29]. EPCR binds PC and APC with high affinity in a Ca^2+^-dependent manner through the Gla domain of these ligands. EPCR is considered a cofactor of protein C as it accelerates TM-thrombin complex-mediated activation of PC to APC by concentrating protein C near the surface of the vessel wall. APC regulates thrombin production. Therefore, after being activated, APC dissociates from the complex with EPCR and takes part in degradation of cofactors Va and VIIIa, thus finally down-regulating further thrombin formation (anticoagulant mechanism) (Figure 1) [30]. 

EPCR is localized in small amounts within the recycling compartment of the cytoplasm, where regulation of its activation occurs via circulation of the EPCR-PC/APC complex between the cell surface and recycling compartment of the endothelial cells. It has been suggested that internalization may promote clearance of PC/APC ligands from circulation [31]. 

Other serine proteases bind to EPCR with similar affinity as PC, and also in a Ca^2+^-dependent mechanism. These include factor VIIa (FVIIa) and factor Xa (FXa) that are involved in hemostasis, tissue repair, inflammatory processes and cancer dissemination (Table 1) [14,32,33].

New EPCR ligands have been discovered and include *Plasmodium falciparum* erythrocyte membrane protein, and a specific variant of the T-cell receptor present on a Vδ2neg γδ T cells [2].

There is substantial evidence from in vivo studies that EPCR plays a notable role in anticoagulation. EPCR inhibition can increase the thrombotic state of animals, causing vessel occlusion in mice. Similarly, the presence of autoantibodies against EPCR was described in patients experiencing episodes of thrombosis [34].

APC/EPCR activation results in cytoprotective, anti-inflammatory and anti-apoptotic cellular effects that have been widely described in multiple injury models, as well as inflammation and ischemic stroke models [20,35,36,37,38]. It has been demonstrated with in vivo studies that the brain is particularly sensitive to APC-induced signaling. There APC exerts anti-apoptotic and neuroprotective effects resulting in decreased brain ischemia [37,38]. APC may be generated in human brain as APC levels appear to decrease in stroke patients [37]. Clinical research has focused on the APC variant, 3K3A-APC, that is cytoprotective and independent of the canonical anticoagulant activity induced by activation of PAR-1 [37,39]. Neuroprotective therapy with recombinant 3K3A-APC is being evaluated in ongoing National Institutes of Health (NIH)-funded clinical trials for ischemic stroke [38]. Additionally, the anti-inflammatory and cytoprotective features of APC/EPCR interactions were exploited to treat patients with severe sepsis in the PROWESS clinical trial [36,37,40].

The EPCR-mediated role in vascular barrier function and cytoprotective features in endothelium may potentially contribute to cancer development and progression. Various cancer cells express the EPCR (e.g., colorectal cancer, lung cancer, malignant pleural mesothelioma, breast cancer, ovarian cancer, gastric cancer) [41,42,43,44,45,46]. Upregulation of EPCR in malignant cells results from gene amplification and DNA hypomethylation [41]; however, its distribution varies. In studies of gynecological cancers, EPCR was absent from microparticles derived from the ovarian adenocarcinoma cell line (OVCAR-3), but was detected on intact OVCAR-3 cells [46]. The expression of EPCR and key proteins associated with EPCR-dependent signaling (protein S, protein C, thrombomodulin, Factor V, VIII and PAR-1 and PAR-2) was investigated in endometrial and ovarian cancers [47] and compared with benign tumors. Interestingly, the gynecological cancers expressed EPCR and associated proteins, but to a lesser extent than benign tumors. PAR-1 expression did not differ between benign and malignant tumors.

The clinical significance of the APC/EPCR complex in cancer biology appears to vary depending on the tumor subtype (Table 2). Animal model-based studies revealed that increased expression of EPCR in cancer cells inhibits proliferation and migration through the extracellular signal-regulated kinase, ERK/AKT-dependent signaling pathway [41,44] and promotes apoptosis via BAX and BCL2 factors, thereby limiting tumor growth and dissemination [11,44]. Transgenic mice overexpressing EPCR (Tie2-EPCR) had a 50–92% decrease in liver and lung metastases compared with wildtype animals. Additionally, treatment of the mouse with recombinant human APC or a signaling-proficient mutant, APC-2Cys (with reduced anticoagulant activity), also led to a reduction of metastatic melanoma foci by inhibiting transendothelial migration of malignant cells [11,48]. The APC/EPCR interaction, e.g., in melanoma cells, likely decreases expression of endothelial adhesion molecules, such as P-selectin that is essential for tumor cell-endothelium interactions during extravasation, ultimately hindering dissemination [11]. 

Concurrently, and in contrast with the data that APC and EPCR are tumor suppressors, there are multiple studies that demonstrate EPCR expression by cancer cells can actually promote growth and dissemination as a result of antiapoptotic effects [13,45,49]. This has been described in lung cancer cells in vitro, where the APC/EPCR complex inhibits apoptosis by triggering the AKT/ERK signaling pathways [50]. Furthermore, in vivo experiments where the APC/EPCR interaction was blocked resulted in reduced metastasis. There appears to be an association between high levels of EPCR and poor prognosis in lung cancer patients, especially at early stages of the disease. 

Experiments with human and murine xenograft breast cancer models revealed that EPCR silencing decreased primary tumor growth and the establishment of metastatic foci in distant organs, e.g., bones and lungs. Surprisingly, EPCR stimulation by APC under both in vitro and in vivo conditions did not influence these effects. It has been suggested that EPCR-mediated matricellular secretion of proteoglycan SPOCK1/testican-1 was responsible for inducing breast cancer growth. Analysis of the EPCR transcript in 286 breast cancer patients revealed a correlation between high EPCR levels and patient survival [45]. EPCR (PROCR, protein C receptor, CD201) expression was also assessed in invasive ductal carcinoma of the breast from 271 patients with stage II or III disease. Positive expression of EPCR correlated with development of metastases and decreased disease-free and overall survival [51].

Keshava et al. [52] demonstrated that the role of EPCR in tumor growth varied depending on the time breast cancer cells had been injected into the mammary fat pad. Namely, malignant cells positive for EPCR expression exhibited greater tumor growth than EPCR-negative cells, but only during the first 40 days of implantation into the mouse. Interestingly, after that time the growth of those tumors derived from EPCR-positive cells was slower than that from control cells, i.e., EPCR-negative, such that 60 days post injection the tumor volume of EPCR-positive cells was about 30% smaller than tumor originating from control cells. Moreover, animals bearing EPCR-negative tumors presented with more advanced angiogenesis, swollen lymph nodes and extensive inflammatory reactions on the skin, with some animals exhibiting ulceration above the tumor. Necrosis, a marker of more aggressive breast cancers, was also noticed only in EPCR-negative tumors. None of the EPCR-positive tumors demonstrated skin ulceration and angiogenesis was less advanced than in control tumors. EPCR expression was eventually lost in some tumors that were primarily composed of EPCR-positive cells, which confirms the complexity of EPCR-dependent signaling in tumor progression, and also indicates its potentially protective role in preventing cancer growth and dissemination. 

Another study that utilized two breast cancer cell lines, MDA-MB-231 and MDA-MB-435, unveiled that APC induced chemotaxis and invasion through the EPCR/PAR-1-dependent signaling pathway [49]. The aggressive, triple-negative breast cancer subtype has been shown to express high levels of EPCR, which is correlated with tumorgenicity [53]. The expression of EPCR was also demonstrated on breast cancer stem cells and, interestingly, this population of cells could initiate tumors [53]. Complete inhibition of APC-mediated effects could be achieved with antibodies directed to EPCR and PAR-1. Additionally, the role of APC in angiogenesis was discovered by APC stimulation of MDA-MB-231 breast cancer cells, which resulted in endothelial tube formation [4]. The increased proliferation of endothelial cells in vitro and induced angiogenesis in animal models were regulated by APC/EPCR/MAPK and partly by the PAR-1 signaling pathway [54]. An inhibitor of APC, PCI, reduced tumor cell invasion in vitro by blocking protease activity [13]. 

There are conflicting data regarding the association between EPCR expression and chemosensitivity in tumors. In lung cancer, expression of EPCR has been proven to increase chemosensitivity, while in colorectal cancer such effects were not observed as the overexpression of EPCR was accompanied by expression of neighboring chemoresistance genes on chromosome 20q [41]. Chemotherapy itself, e.g., with doxorubicin, may cause down-regulation of EPCR in endothelial cells [55]. There is a case report that describes a cancer patient receiving chemotherapy who was deficient in APC and who subsequently developed clinically significant thrombosis [56]. 

## 3. PAR-1 and Cancer

Protease activated receptor-1 (PAR-1) is one of four isoforms identified to date. It is a G-protein-coupled receptor, and as with EPCR it is located in the lipid raft compartment of the cell membrane. PAR-1 is composed of seven transmembrane α-helices, a cytoplasmic domain for G-protein coupling, and an extracellular N-terminus. A comprehensive review of PARs was published in 2015 [16]. The present review focuses on matters pivotal for understanding the role of PAR-1 in APC/EPCR/PAR-1-associated signaling. PAR-1 expression was documented for nearly all cell types in the blood vessel wall (ECs, fibroblasts, myocytes), and blood (platelets, neutrophils, macrophages, leukemic white cells) with exception of erythrocytes. It was also identified on epithelium, and in neurons, astrocytes and immune cells [16]. Importantly, PAR-1 expression was also detected in multiple cancer subtypes, including epithelial carcinomas, melanoma, glioblastoma (GBM), giant cell tumors and sarcoma, and is present on both malignant cells as well as on tumor-associated stromal components (reviewed in [16,57]). The latest findings indicate that PAR-1 expression occurs on extracellular vesicles derived from cancer cells, e.g., prostate, breast, pancreas [58,59]. Antibodies to PAR-1 have also been discovered in cancer patients [60].

There are multiple PAR-1 ligands (Table 1). Among these thrombin, APC and haemostatic factors VIIa, Xa and their complex with TF (TF-VIIa-Xa) have been proven to be associated with EPCR and PAR-1-dependent signaling. 

Importantly there are canonical (classical) and non-canonical modes of PAR-1 activation. The main activator of PAR-1, thrombin, represents the canonical signaling pathway. After binding to the receptor N-terminal sequence LDPR41-S42 the R41-S42 peptide bond becomes cleaved thereby generating a new sequence referred to as a tethered ligand of PAR-1. The tethered ligand binds residues 42SFLLRN47 in the conserved region of the second loop of the receptor to induce transmembrane signaling [61]. Interestingly thrombin-mediated PAR-1 activation does not require the presence of any cofactors due to highly acidic regions (P4-L38 and P2-P40 residues) that increase thrombin affinity and facilitate proper cleavage of PAR-1. However, for APC to cleave the N-terminus of PAR-1 in cancer cells, EPCR is required as a cofactor [62]. PAR-1 activation mediated by thrombin causes receptor coupling to Gα protein (Gαq, Gαi and Gα12/13) and Gβγ. Heterotrimers composed of PAR-1 and Gαq lead to activation of MAP kinase (mitogen activated protein kinase) and increased Ca^2+^ concentration, while complexes of PAR-1 with Gα12/13 activate the small G-protein, RhoA (Ras homolog gene family member A) [63]. Activation of PAR-1 by APC in turn leads to activation of Ras-related C3 botulinum toxin substrate 1 (Rac1).

Finally, thrombin-mediated PAR-1 activation drives production of cytokines, chemokines, growth factors and bioactive lipids to trigger inflammation, adhesion, and endothelial barrier disruption, all of which contribute to tumor growth and survival. It also promotes angiogenesis and transendothelial migration resulting in tumor progression and metastasis [57]. Studies done in vitro showed that overexpression of PAR-1 in cancer cells is associated with greater invasiveness and ability to disseminate, making PAR-1 expression in some cancers an unfavorable prognostic factor in terms of overall survival or local recurrence. 

To complicate matters, a recent study with a pancreatic ductal adenocarcinoma cell line surprisingly demonstrated that PAR-1-associated signaling instead limits tumor growth, probably via induction and maintenance of a mesenchymal-like cell phenotype [64]. This indicates that there are still unexplored molecular events associated with PAR-1 signaling pathways.

Ligands other than thrombin (e.g., MMP-1, APC, FXa) can activate PAR-1 in noncanonical ways resulting in biological effects that differ from those driven by thrombin. Thrombin-mediated PAR-1 activation increases endothelial permeability, while the APC-mediated effects result in endothelial barrier protection and in some cancer models are antimetastatic. MMP-specific signaling patterns exhibited by PAR-1, known as biased agonism, also result in distinct functional outputs compared to thrombin-induced PAR-1 activation. Cleavage of the extracellular portion of the PAR-1 receptor by thrombin occurs at a canonical R41-S42 site, while MMP-1 cleaves PAR-1 at a novel site (D39-P40) resulting in a tethered ligand that is two amino acids longer (PR-SFLLRN) than that generated by thrombin. Subsequent peak Akt signaling occurs after five minutes in thrombin-triggered activation, while MMP-1-triggered Akt activation occurs after one hour and induces different cellular events [65].

Noncanonical PAR activation has also been documented for FXa, which leads to tunica intima endothelial receptor tyrosine kinase 2 (Tie2) activation in an EPCR-dependent manner, and ultimately endothelial barrier enforcement by upregulation of zona occludens 1 (ZO-1) to stabilize cell-cell junctions. 

## 4. EPCR and PAR-1 Interactions

### 4.1. G-Proteins

**Figure 2:**
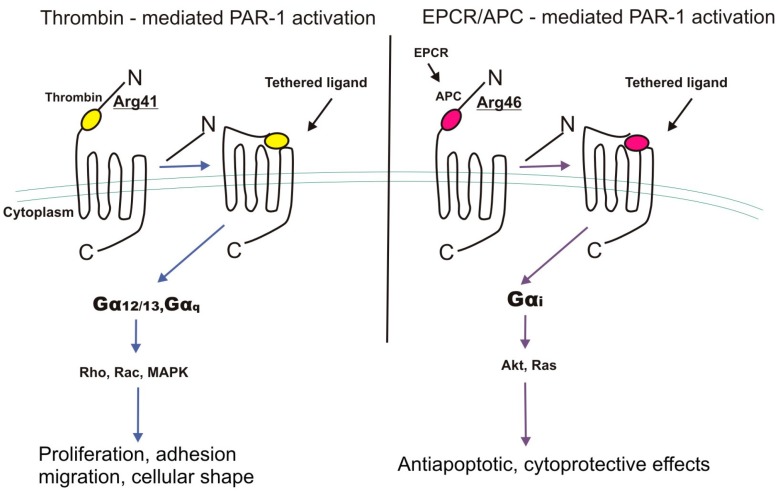
Distinct mechanisms of PAR-1 (protease-activated receptor 1) activation by thrombin- and activated protein C (APC)/endothelial protein C receptor (EPCR) complex resulting in different biological effects. MAPK, mitogen activated protein kinase, proteins Rho, Rac, Akt, Ras.

There is evidence that most cellular effects of EPCR-bound APC require PAR-1 activation [66]. APC is much less effective at cleaving and activating PAR-1 compared to thrombin [67] and it also evokes different biological effects [68]. Classic thrombin-dependent proteolysis of PAR-1 at canonical Arg41 couples G12/13 and Gq signaling that ultimately, through MAPK and RhoA activation, elicits proinflammatory effects. Interestingly, the APC/EPCR complex causes the dissociation of EPCR from caveolin-1 in lipid rafts resulting in PAR-1 cleavage at non-canonical Arg46 and further activation of protein Gi instead of G12/13 and Gq [32]. Finally, β-arrestin-mediated phosphorylation of Akt leads to activation of Ras-related C3 botulinum toxin substrate 1 (Rac1) signaling. APC-cleaved PAR-1 remains present on the cell surface for some time likely blocking thrombin-mediated PAR-1 activation. The slower and longer activation of PAR-1 by APC compared to thrombin may explain the up-regulation of more cytoprotective and anti-inflammatory genes (Figure 2). 

For example, there is increased expression of the sphingosine-1-phosphate receptor, which upregulates Tie2, the Angiopoietin-1/Angipoietin-2 ratio and VE-cadherin to ultimately reinforce vascular barrier protection [11,69]. Regulation of cytoskeleton-mediated cell-cell interactions and blocking of actin stress fiber formation is observed as a result of APC-mediated PAR-1 activation [70,71]. The cytoprotective properties of the APC/EPCR/PAR-1 signaling cascade and enhancement of endothelial barrier inhibits tumor cell extravasation and dissemination as a result of the PAR-1-dependent mechanism. Absence of endogenous APC as a result of loss of VE-cadherin junctions increases vascular leakage and cancer cell extravasation. The anti-inflammatory effects of EPCR/PAR-1 interactions are associated with suppression of the nuclear factor kappa light-chain enhancer of activated B cells (NF-kB) signaling pathway [38,66]. Cellular models confirmed that blockade of either one of these receptors (EPCR, PAR-1, S1P1) eliminated the protective effect of APC. The role of APC/EPCR/PAR-1 pathway activation was also described in keratinocyte proliferation [72]. The co-localization of EPCR and PAR-1 within lipid compartments and caveolae microdomains in endothelial cells plays a role in interactions with PC. Recent experiments demonstrated that administration of PC to septic mice exerted anti-inflammatory and anti-apoptotic effects in the septic lungs and also resulted in reduced caveolin-1 expression in the lungs, thus facilitating the interaction between EPCR and PC that induced PAR1-dependent cytoprotective signaling.

Additionally, it has been suggested that APC light chain amino acid residues outside the EPCR-binding site enable activation of APC-mediated PAR-1 cytoprotective functions [73]. There are molecules termed “parmodulins” that may interact with the cytosolic face of PAR-1 and cause similar APC-like, PAR-1-dependent cytoprotective signaling in endothelium [74].

There are contradictory findings that are of paramount importance with respect to the role of the APC/EPCR/PAR-1 axis in cancer progression [42,75,76]. Wang et al. [42] demonstrated that gastric cancer tissue expresses elevated levels of EPCR and that EPCR-mediated APC activation induces proliferation and migration of the MGC803 gastric cancer cells. Additional experiments showed that the EPCR/APC/PAR-1 cascade also induces angiogenesis in gastric cancer cells [76]. The microvessel density (MVD) of 61 surgically removed primary gastric cancer tumors were assessed by using endothelial markers CD31 and CD34. Reportedly the mean value of MVD was higher in gastric cancer tissues expressing EPCR compared to EPCR-negative tissue. Human umbilical vein endothelial cells (HUVECs) were cultured with tumor-conditioned medium derived from EPCR knockdown or PAR-1-inhibited MGC803 gastric cancer cells. Interestingly, knocking down EPCR by small interference RNA and blocking PAR-1 activity by antibody resulted in inhibition of phosphorylation of extracellular signaling-regulated kinases 1 and 2 (ERK1/2) and Akt, thereby leading to suppression of the proliferation, migration and tubule formation potential of HUVECs. These findings indicate that EPCR and PAR-1 reciprocal interactions contribute to cancer-promoting events in malignant cells [42]. 

### 4.2. B-Arrestin

Although some mechanisms of triggering protein Gi instead of G12/13 and Gq as a result of APC-EPCR/PAR-1 interactions explain the cytoprotective role of PAR-1, it still remains unclear whether Gi is totally responsible for these effects. There are interesting data that explain the phenomenon of PAR-1 activation by two different proteases. The PAR1-induced cytoprotective activity of APC may not be mediated through either one of the G-proteins, but rather via β-arrestin-2-biased signaling [77]. It has been demonstrated that APC occupancy by EPCR leads to the recruitment of G-protein-coupled receptor kinase 5 (GRK5) to the plasma membrane and phosphorylation of the PAR-1 cytoplasmic domain. This phosphorylation suppresses PAR-1-dependent activation of G proteins resulting in β-arrestin-2 biased PAR-1 cytoprotective signaling independent of the protease cleavage site and protein Gi coupling. Interestingly, thrombin also exerts PAR-1-dependent cytoprotective effects by β-arrestin-2-induced recruitment of disheveled 2 (Dvl-2) scaffolding protein and Rac1 signaling. 

### 4.3. Factor VII/VIIa and Factor Xa (FXa)

**Figure 3:**
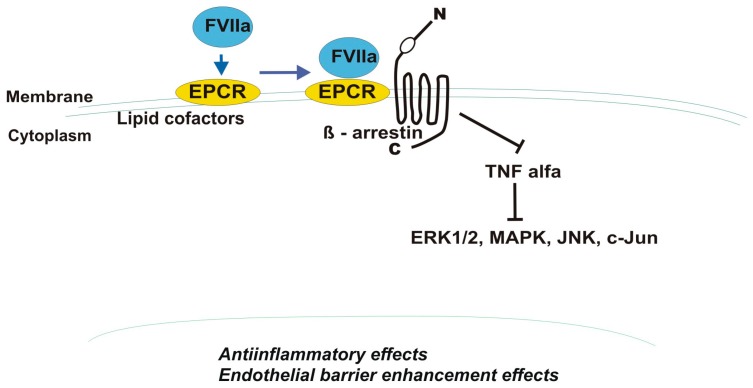
Factor VIIa—mediated activation of endothelial protein C receptor (EPCR) and protease-activated receptor 1 (PAR-1) leading to suppression of tumor necrosis factor alfa (TNF-α)—dependent signaling. ERK—extracellular signal-regulated kinase; MAPK—mitogen activated protein kinase, c-Jun, JNK—N-terminal kinase.

Other serine proteases that may occupy EPCR in a Ca^2+^-dependent mechanism and exert protective effects on the endothelial barrier are factor VII/VIIa and factor X/Xa (FXa), which bind EPCR through the 4-carboxyglutamic acid domains [14,33,78]. The cytoprotective properties mediated by FVIIa binding to EPCR depend on PAR-1 and β-arrestin-1 activation (Figure 3). FVIIa-treated ECs in vitro had reduced expression of cellular adhesion molecules and adherence of monocytes to ECs. These effects were dependent on PAR-1-mediated suppression of tumor necrosis factor α (TNF-α). PAR-1 antibodies or small interfering RNA abolished TNF-α-induced activation of ERK1/2, p38 MAPK, JNK, NF-κB, and C-Jun factors as well as lipopolysaccharide (LPS)-mediated in vivo inflammatory responses in lungs of mice overexpressing EPCR. Additionally, FVIIa in the PAR-1/MAPK/Rac1-dependent pathway has been shown to reduce LPS- as well as vascular endothelial growth factor (VEGF)-induced vascular leakage thereby revealing FVIIa barrier-protective features [79,80]. EPCR serves as a receptor for FVIIa on endothelial cells and has recently been found to bind FVIIa on human platelets, which have been widely described to play a role in cancer progression [81,82]. PAR-1 cleavage by FXa is also highly enhanced in the presence of EPCR, which, similar to APC, dissociates from caveolin-1 after FXa binding [78]. Unfortunately, FVIIa and FXa/EPCR-mediated PAR-1 activation is not well explored in cancer tissues.

### 4.4. Tissue Factor

Tissue factor is the main procoagulant protein expressed on cancer cells, and is responsible for thrombin generation in the tumor microenvironment independently of blood coagulation. Thrombin is pivotal for APC activation. Apart from thrombin generation, TF plays a role in EPCR-mediated PAR-1 activation via complex formation with coagulation factors TF/VIIa/Xa, and is regarded as an EPCR cofactor [33]. The activation of p44/42 MAPK signaling induced by TF/FVIIa/FXa on activated endothelial cells is EPCR-dependent [83]. 

There is evidence that APC/EPCR/PAR-1 cooperate in the up-regulation of TF to increase cancer cell invasiveness [59]. APC also mediates degradation of TFPI, an inhibitor of TF-dependent coagulation, and through this “procoagulant” pathway surprisingly regulates the pro-metastatic potential of thrombin generated due to TF expression on cancer cells [84].

The complexity of APC/EPCR/PAR-1 and TF interactions was shown in an animal model of malignant pleural mesothelioma (MPM) [52]. Intrapleural injection of mesothelioma cells expressing PAR-1 and TF, but that were EPCR negative, led to larger tumor growth, while ectopic expression of EPCR in aggressive mesothelioma cells attenuated their proliferative potential. Interestingly, EPCR inhibition in non-aggressive MPM cells that overexpressed TF increased their tumorgenicity, thus demonstrating the protective role of EPCR in cancer promotion. 

### 4.5. Thrombomodulin, TM

Thrombomodulin is a tumor suppressive protein that when expressed in several types of cancer cells decreased tumor invasiveness and improved patient survival [85,86]. The level of soluble TM shed from cancer cells to the plasma correlates with disease advancement and is associated with poor prognosis [87,88]. Expression of TM on cancer cells was associated with better differentiation in patients with squamous cell carcinoma of the oral cavity [88]. Furthermore, TM decreases PAR-1 and NF-κB activation and significantly suppresses pancreatic cancer tumor growth [89]. 

### 4.6. Hematopoietic Stem Cells

Gur and Cohen [7,8] demonstrated that long-term repopulating hematopoietic stem cells (LT-HSCs) originating from adult murine bone marrow (BM) and that expressed EPCR and PAR-1 had the highest repopulation and self-renewal potential. The EPCR-positive LT-HSCs accumulate in close proximity to BM endothelial subpopulations expressing elevated thrombomodulin, which in the presence of thrombin induces APC-mediated PAR-1 activation. EPCR/PAR-1 signal activation and restriction of nitric oxide (NO) production induces LT-HSC BM repopulation, retention, and chemotherapy resistance to induce anti-apoptotic effects and prolong cell survival. 

PAR-1-associated signaling in LT-HSCs also results in their recruitment to the blood circulation and allows them to retain developmental potential. The PAR-1-dependent signaling pathway results in nitric oxide (NO) reduction leading to Cdc42 downregulation and increased adhesion associated with VLA4. The integrin VLA4 binds fibronectin and VCAM-1 and is crucial for CXCL12/CXCR4-mediated BM HSC retention. The balance between retention of LT-HSCs in BM or trafficking to the blood is dependent on PAR-1 activation by APC or thrombin, levels of NO, and surface CXCR4 expression. 

Additionally, the thrombin-PAR-1-activated signaling cascade plays a role in BM cell trafficking under conditions of stress or inflammation, when increased levels of thrombin are generated and rapidly enter the BM. This induces PAR1-dependent hematopoietic stem and progenitor cell mobilization, motility and recruitment of LT-HSCs into the bloodstream. 

Summing up, along with pro- and anticoagulant signaling, which has also been widely described for malignancies, factors of the coagulation system cooperate within the bones in non-hemostatic pathways to regulate bone-remodeling processes, stem cell interactions, BM cell retention or release to the blood stream during both steady-state and inflammatory conditions. The expression of PAR-1 has been detected in acute myeloid leukemia cells as well as leukemia stem cells [90]. Therefore, the hypothesis that EPCR and PAR-1-dependent signaling is implicated in development of hematopoetic malignancies is justified, especially in light of the fact that myeloid cells such as osteoclasts express TF and prothrombin.

### 4.7. Microbiome and EPCR/PARs Interactions

Microbial factors and infection-associated inflammatory processes have aroused great interest with respect to carcinogenesis. Proteases are essential for the normal functioning of bacteria and may initiate pathological events such as increased enteric permeability and inflammation by acting through protease receptors [91]. *Streptococcus pyogenes* and *Staphylococcus aureus* on the skin induce inflammatory reactions by producing proteases that activate PARs on keratinocytes [92]. Epidemiologic studies have reported an association between periodontitis and *Porphyromonas gingivalis* (Pg) infection with increased risk of oral squamous cell carcinoma (OSCC) and orodigestive cancer death [93]. Interestingly Pg promotes an oral squamous cell carcinoma through the PARs-activated signaling pathway that results in increased MMP9 expression and thus cancer invasion [94]. The newest study reported that Pg induces host molecular changes that promote EMT (epithelial-mesenchymal-transition), a process highly regulated by PAR-1 activity in cancer cells and responsible for dissemination [95,96]. 

There are reports that EPCR is also involved in EMT among mammary stem cells, a subpopulation of cells determined to have greater invasive potential [97,98]. Further studies are needed to explore the role of the APC/EPCR/PAR-1 cascade in this part of the metastatic process.

Increased permeability of endothelium or intestine as a result of the action of bacterial proteases is an event similar to that induced by metastasizing cancer cells for dissemination. That is why the findings that the same receptors are involved in both processes are so exciting and require extensive investigation [91,92,93,94]. 

The cyclic dipeptide with documented anti-inflammatory and anti-cancer activity that is naturally produced by bacteria, fungi, marine sponges, gorgonian and red algae has been reported to inhibit tumor necrosis factor (TNF)-α, and interleukin (IL)-1β-induced EPCR shedding in human umbilical vein endothelial cells (HUVECs) [99].

### 4.8. EPC/PAR-1 and Neurons

The altered nerve function orchestrated by PAR-1-dependent signaling in the central nervous system and in peripheral nerves has been intensively investigated as an important pathogenic factor of many inflammatory and neurodegenerative diseases [100,101]. The influence of inflammation in cancer is undeniable. Thus the molecular connections between neurotoxicity and malignancy are interesting [102]. Data show that neurons within the inflamed central nervous system and peripheral tissues (lung, intestine) overexpress PAR-1, and in this way are exposed to neurotoxic inflammatory mediators resulting in brain atrophy or enteritis [99,100,103].

Neurons are another cell population where the activation of PAR-1 either by thrombin or the anticoagulant APC has differential effects [104]. It has been documented that thrombin/PAR-1/N-methyl-d-aspartate receptors (NMDAR) regulate long-term potentiation (LTP) in the hippocampus thus causing persistent strengthening of synapses and production of long-lasting increases in signal transmission between neurons. Activated protein C may increase synaptic plasticity in a mechanism that requires the activation of S1P1R and intracellular Ca^2+^ stores. These APC/EPCR/PAR-1-mediated effects on hippocampus functions may be useful in the therapy of neurodegenerative disease and may also be due to antiapoptotic effects exerted by APC in neurons [105]. Moreover, there are findings that APC reduces the nuclear level of NF-κB p65 in hippocampal neurons from glutamate-induced excitotoxicity via binding to EPCR and subsequent PAR-1-activated signaling [106]. Although there is a lack of direct evidence of APC/APCR/PAR-1-dependent signaling in brain tumors the findings of their reciprocal interactions in neuronal tissue suggest that such processes may exist. 

## 5. Treatment

The fact that the results of in vitro and in vivo cancer models assessing the role of APC/EPCR/PAR-1 in tumor progression are contradictory makes the discoveries of therapeutic drugs very difficult. The basic question of whether there are inhibitors or activators of the APC/EPCR/PAR-1 cascade that inhibit cancer development and dissemination remains unanswered.

The APC/EPCR/PAR-1 axis has been demonstrated to reduce organ damage in models of multiple pathologies like stroke, sepsis and autoimmune disease so that a clinical trial (NCT00533546) with APC treatment in ischemic stroke has been initiated (http://www.clinicaltrials.gov). Moreover, APC has been approved by the Food and Drug Administration (FDA) for the treatment of severe sepsis due to results from the PROWESS clinical trial that drotrecogin alfa (DrotAA), recombinant human APC, reduces mortality in patients with severe sepsis [36,37,40,107]. Although DrotAA has been withdrawn from ischemic patient therapy, the clinical trials with APC variants (e.g., 3K3A-APC) are conducted based on cytoprotective, not anticoagulant (like with DrotAA) properties of APC [108]. 

Based on the cytoprotective effects exerted by APC in the above-mentioned diseases it has been suggested that exogenous APC administration might also serve as a new therapeutic option for cancer patients to protect from or treat metastasis. Unfortunately, the very short half-life of APC, approximately 15-min, requires continuous infusion of the drug, which may be problematic for cancer patients. 

Additionally, the bleeding complication frequently observed during APC therapy may limit use of this drug in practice. The in vitro and in vivo studies that demonstrate the potential of APC and PAR1-based peptides (e.g., TR47 given at a dose of 125 μg/animal) to enhance endothelial barrier function, to decrease transendothelial migration of malignant cells, and finally reduce metastasis to distant organs, has stimulated a search for activators of the APC/EPCR/PAR-1 axis that might be a valuable therapeutic approach to prevent metastasis.

It is now accepted that thrombin facilitates the interaction between tumor cells and platelets and endothelial cells, thereby enabling malignant cells to seed and metastasize. Therefore, this is an important target for therapeutic research [109]. Dabigatran, a thrombin inhibitor, delivered in combination with gemcitabine, inhibited primary tumor growth and prevented tumor cell dissemination from pancreatic cancer in mice [110]. Additionally, there are data that inhibition of APC/EPCR or the PAR-1 cascade is effective for killing cancer. Inhibition of the thrombin-induced PAR-1/Rho GTPase signaling pathway activation by ALEX1 (a novel tumor suppressor gene, and member of the armadillo family) limits gastric cancer metastasis [1]. Importantly, the expression of ALEX1 may be decreased in a variety of solid tumors correlated with clinical features, such as cell differentiation and staging. The anticancer activity of some chemotherapeutic agents has also been shown to be associated with membrane receptors [111]. Namely, doxycycline inhibits breast cancer migration and metastatic potential through the PAR-1/NF-κB/miR-17/E-cadherin pathway. 

The newest study reporting the inhibitory role of PAR-1 in pancreatic ductal cancer growth and all studies presenting an inhibitory role for EPCR in cancer progression suggests that the contribution of APC/EPCR/PAR-1 signaling in cancer biology differs between tumor subtypes. Thus, targeting should be applied with care as this area still remains unexplored.

## 6. Conclusions

The interactions between EPCR and PAR-1 are complex and pleiotropic. Understanding of the mechanisms induced by these receptors improved considerably over the last several years, which creates a chance for future pharmacological interventions that affect receptor system. 

## Figures and Tables

**Table 1:** Ligands leading to EPCR (endothelial protein C receptor) or PAR-1 (protease-activated receptor-1) activation [2,16]. APC (activated protein C), TF (tissue factor), MMPs (matrix metalloproteinases).

Receptor	EPCR	PAR-1
Ligand	Factor VIIa Factor Xa TF-VIIa-Xa TF-VIIa *Plasmodium falciparum* erythrocyte membrane protein T-cell receptor present on a subset of Vδ2neg γδ T cells	Thrombin Factor Xa TF-VIIa-Xa APC Plasmin Granzyme A Gingipains-R Trypsin MMP-1, -9, -2, -13, -14

**Table 2:** Distinct cellular effects mediated by endothelial protein C receptor (EPCR) in different cancer settings.

Cancer Cell Line/Xenograft	Mechanism	Cellular Effects
Colorectal cancer [41]	ERK/AKT-dependent signaling	Inhibition of migration
A375 melanoma cells B16F10 melanoma cells [11,48]	ERK1/2—dependent signaling	Reduction of metastatic foci by inhibition of transendothelial migration
Malignant Pleural Mesothelioma [44]	ERK/AKT-dependent signaling; BAX, BCL2 factors	Inhibition of proliferation and migration Promotion of apoptosis
MGC803 gastric cancer cells [42]	ERK1/2—dependent signaling	Increased proliferation and migration
Breast cancer cells line [43,45,49,52]	SPOCK1/testican 1-mediated signaling MAPK-signaling	Increased expression of integrins, proliferation, 3D tumor growth and cells survival Angiogenesis
Lung adenocarcinoma [50]	ERK/AKT-dependent signaling	Inhibition of apoptosis
